# Review of Dr. Allport’s Proposition to Separate the Mechanical from Other Departments of Dental Practice

**Published:** 1875-02

**Authors:** J. Richardson

**Affiliations:** Terre Haute, Ind.


### DENTAL REGISTER.

VOL. XXIX.] FEBRUARY, 1875. [No. 2.

### REVIEW OF DR. ALLPORT'S PROPOSITION TO SEPARATE THE MECHANICAL FROM OTHER DEPARTMENTS OF DENTAL PRACTICE.

BY J. RICHARDSON, TERRE HAUTE, IND.

"Now, in the names of all the gods at once,
Upon what meat doth this our Cæsar feed,
That he is grown so great."—*Shak.*

In an address recently delivered before the American Academy of Dental Science, Dr. W. W. Allport earnestly and elaborately advocates the above proposition. The position taken by him is, if we comprehend it rightly, that the practice of what are called the higher branches of dentistry, as the operative, medical and surgical, is established upon a medical basis, and therefore allies these branches closely to medical science. while what is known as the mechanical branch, or dental prosthesis, relates only to a mechanical art, requiring but a limited knowledge of medical science,—that being thus essentially dissimilar, incongruous and incompatible, they should be divorced. This, we believe, is a fair epitomized statement of his proposition.

It might be reasonably expected that so extraordinary a proposition as that embodied in this address—a proposition formally and ostentatiously submitted to the deliberate and critical judgment of an intelligent profession through the medium of a prominent organization which deemed it of sufficient importance to perpetuate it in pamphlet form, should be fortified by well grounded considerations of policy, expediency, propriety and necessity. We concede to the doctor's treatment of his subject a certain plausibility which is well calculated to mislead a casual reader or careless thinker, but the arguments employed in support of his scheme will not, we believe, bear the test of even a moderately searching analysis. If this shall prove to be true, we will do our distinguished friend the justice to concede that the misfortune of failure will not rest so much with himself as in the inherent weakness and untenableness of the proposition itself.

In attempting to review this rather remarkable contribution to dental literature, we are confronted at the outstart by a brace of difficulties, one of which is to treat the subject-matter of it, as we sincerely desire to do, with gravity, and the other is to resolve some sort of order out of the doctor's disjointed methods of reasoning, for our friend scatters his shot like a blunderbuss, projecting them here and there into a mass of verbiage in the most reckless and promiscuous manner, and without any christian regard to continuity of arrangement. How many of these comparatively harmless little missiles may be found to have penetrated beneath the cuticle of his subject, it is our present purpose to ascertain.

"The true mission of medical science," we are told, "is to preserve and restore health, and save life and limb, and not to make or have to do with the making of artificial substitutes any further than as they shall be made directly useful in subserving these purposes." A truer and more comprehensive definition would be that which should harmonize with the

Baconian philosophy, that the grand and ultimate purpose of all the natural and physical sciences is to contribute something to the sum of human happiness, and to promote the welfare of the race. Properly, the preservation and restoration of health, and the saving of life and limb, are but the contributions of medical science to this grand humanitarian object, and nothing may be considered unworthy of its disciples which shall fulfill, in the most perfect manner, this higher mission.

But we will accept, for the purposes of this review, the definition given. If it be so that the true mission of medical science is, amongst other things, to preserve health and save life, then, next to the preservation of the natural teeth, does the substitution of artificial ones fulfill most perfectly this grand mission by contributing to these ends. We need scarcely insult the author's intelligence by reminding him that mastication, as a preliminary act of digestion, exercises a controlling influence over the health of the individual, and that its imperfect performance almost certainly compromises it by engendering disorders of the *primæ viæ*, and oftentimes menaces and prematurely destroys life itself by awakening, through a pervertion of the nutritive functions, latent predispositions to some of the gravest forms of disease. And need we remind him further that this preliminary act can not, in any sufficient degree, be accomplished without the proper organs, natural or artificial. Making allowance for the difference in value of natural and artificial teeth in respect to the efficient performance of this function, the man who skilfully constructs a set of teeth, answering even in a tolerable degree the purposes of the natural organs, meets as fully the claims of the mission here spoken of as any one devoted to the so-called higher branches. If this be true, and we do not think it will be gainsayed, then this intimate and important relation of mechanical practice to health and life, allies it, in the same manner as the other departments, to medical science, and we venture to affirm that there is nothing within the entire range of surgical prosthesis that bears any just comparison to it in the gravity of the personal interests involved.

Following the paragraph already quoted, we are treated to the following sage observation:

"Wig making, and the manufacture of artificial limbs and eyes are useful and respectable callings, and when properly pursued require a good degree not only of mechanical skill, but also of artistic taste, and as well almost might the making of these be taught in the medical college, as the making of sets of teeth form part of the curriculum in a medical specialty."

The facility with which our author confounds things is one of the marked features of his unique production. He here attempts to run a parallel where not the slightest analogy exists, so far as the relation of these several processes to medical science is concerned, and that is the question at issue.

The doctor furnishes us every facility for drawing the line of demarkation between them. He affirms that the true mission of medical science has nothing to do with the making of artificial substitutes only so far as they shall be made useful in subserving the purpose of preserving and restoring health, etc. Now artificial teeth are efficient in the proper mastication of food,—mastication is essential to good digestion, and the latter to a healthful play of the nutritive functions,—therefore artificial teeth are useful in subserving the purposes of preserving health, and of restoring it when previously impaired by reason of defective mastication. Will it be claimed that artificial limbs or eyes have any such relation to the preservation or restoration of health? On the contrary, are they not merely supplemental contrivances for purposes of locomotion and disguise, and which could be dispensed with without prejudice to either health or life.

Does not the author also blunder when he seeks to draw a parallel between the manufacture of artificial limbs, which is but a process incidentally appertaining to a department of general medicine, and the setting of artificial teeth, which is a department in itself. He has sprung a question in this address for the consideration of the profession which contemplates an important modification of its educational policy, and this question must be discussed, therefore, in the light of the fact that dentistry, though in the abstract a department of medicine, is, in the completeness of its educational system, a professional body identical with general medicine. This is not only assumed by its members, but is affirmed by its separate colleges empowered to grant appropriate degrees, its text books embracing every department of its practice, its distinctive journals and multiplied associations, and in the subdivision of its practice into special departments.

Amongst the latter, mechanical practice stands fully and broadly recognized. Certainly Dr. Allport will not claim that the manufacture of artificial limbs is a department of general medicine. Is it not then absurd and wholly inadmissible to claim any analogy between the manufacture of artificial limbs, which is but a process incidentally appertaining to the department of surgery and the setting of artificial teeth which is a department in itself. Had he instituted a comparison between the making of artificial limbs and that of porcelain teeth, he would have hit upon a well defined analogy.

The making of artificial limbs, bearing the same relation to surgery that the manufacture of mineral teeth does to mechanical practice, we must substitute this analogy for that employed by the author, and the sentence, when paraphrased, will then read—"The manufacture of artificial limbs is a useful and respectable calling, etc., but as well almost might the making of these be taught in the medical college, as the making of mineral teeth form part of the curriculum of a medical specialty." We need hardly say that, in this aspect of the matter, the point made by Dr. Allport in this connection loses all force and application as an argument.

The only assignable reason why neither of these processes form any part of the curriculum in medical or dental colleges is that, by common consent, their manufacture has passed out of the hands of special practitioners into those of non-professional parties, not so much because their fabrication was inconsistent with the requirements of the former, as on grounds of greater convenience, reduced cost, and economy of time and labor.

Let us consider this matter a little farther. If a dentist may not, without forfeiting his right to be regarded a special practitioner of medicine, practice the setting of teeth, which has an important relation to the preservation of health, what shall be said of the oculist who "sets" artificial eyes, which have not in the remotest degree any such relation.

The practice of ophthalmology is, in many essential features, much like that of dentistry. There is indeed a marked resemblance in the nature of the demands of these two departments. The primary object and obligation of the oculist is to preserve intact the organs of vision, and secondarily to provide substitutes when the former are lost, just as it is primarily the duty of the dentist to save the natural organs of mastication, and, failing in this, to provide artificial ones. If, by reason of accident, or incurable disease of the eye, this organ is lost, the oculist proceeds by the necessary measures to prepare the socket for the reception of an artificial substitute. He then selects one harmonizing in color, configuration, and expression. This is prepared for easy and comfortable adjustment by grinding, polishing, etc., and then applied. If irritation or inflammation follows its use, he is charged with the duty of combatting these morbid conditions, and all further necessary manipulations of the substitute. Could the analogy between these steps and those employed by the den tist in setting teeth be more striking or complete? Now, if the oculist does not consider it derogatory to his professional standing as a medical specialist to concern himself about all these mechanical details in a matter that is wholly one of personal appearance, need the dentist do so in the construction of an artificial substitute intimately related to the physical well-being of his patient? Is the selection, grinding, polishing, and adjusting of an artificial eye better established upon a medical basis, or more nearly allied to medical science than the setting of artificial teeth? If Dr. Allport had the misfortune to lose an eye, and desired an artificial one, would he not go direct to an oculist instead of the manufacturer of artificial eyes, and if so, would he not be asking the oculist to do that which he here claims would derogate from his own standing as a dentist in the case of artificial teeth? We should have almost as poor an opinion of an oculist who should refuse, under such circumstances, to contribute his professional services in restoring to the countenance of our really handsome friend its usually agreeable expression, as we would of the Dr. should he refuse, on solicitation, to provide the oculist with a set of teeth.

As a further pretext for lopping off mechanical dentistry we are told that it "has, in the last few years, been steadily retrograding, and becoming more and more a trade." In justification of this statement, it is affirmed amongst other things, that, "Up to about twenty years ago the mechanical department of the practice required a practical knowledge of the nature of the precious metals and skill in working them, and a high order of mechanical talent in applying intricate mechanical laws, in fitting and rendering useful the different forms of plates, together with mechanical and artistic skill in adjusting the substitutes so as to subserve the purpose for which they were intended." * * * * And "That plates of precious metal, requiring mechanical skill of a high order to manipulate, have, in a large, majority of cases, been substituted by plates cast from baser metals, or by rubber vulcanized in molds, these requiring neither a high degree of judgment nor mechanical skill to accomplish results tolerably well, limited by the properties of the materials used. "It is to be understood from this, therefore, we suppose, that the character of this department, as a legitimate branch of dentistry, is to be determined, not by the beneficent results of its practice, but by the intricacy, tediousness and complexity of the processes by which such results are obtained." As a curiosity in its way we turn this argument over tenderly for the consideration of the charitably disposed reader with the injunction to "handle carefully" as it is altogether too attenuated and fragile for the purposes of criticism.

Chiefest among the *degenerate* methods mentioned above is that of the rubber base. If this method, amongst others, has degraded the mechanical branch of practice, as alleged by the author, so that it should no longer be considered worthy of alliance with the other departments, is it not rather singular that the profession has, for years past, been engaged in a tedious and vexatious course of litigation, and has expended thousands of dollars to fasten this method upon us by breaking down a patent, the effect of which is to limit its use by the imposition of an onerous royalty. These contributions of effort, time and means have not alone been made by the inferior class of practitioners into whose hands the author alleges this branch has fallen, but chiefly by those of the profession of whom Dr. Allport is a conspicuous representative. We have no doubt but that the Dr. himself has contributed liberally to this purpose, and if he has so voluntarily aided in perpetuating a process which he distinctly affirms has degraded an important branch of his calling, he must pardon us if we apply to him the familiar adage, that "it is a dirty bird that befouls its own nest."

But we deny emphatically that this department has retrograded as charged. Let us contrast briefly the methods now in vogue, with those employed before the introduction of rubber as a base, and in doing so we shall take gold and rubber bases as the representatives of the old and new. We will not include continuous gum work, for it has never been used except in a very limited way, and probably little more twenty years ago than now.

That there is greater economy of time and labor in the use of rubber as a base, no one will deny. That it is more susceptible of accurate adaptation to the mouth is equally clear. By the old method of swaging, a practical adaptation was often difficult and sometimes impracticable by reason of the unavoidable contraction of metallic dies, and the springing or warping of the piece in the process of heating and soldering. The dental journals fairly groaned under the load of contributions suggesting ingenious devices, and complicated methods for obviating these troubles. In the use of rubber there is perfect exclusion of foreign substances subject to decomposition, and consequent cleanliness and purity. By the older methods, the utmost care in adjusting the teeth to the base and to each other was inadequate to avoid interstices for the lodgment of pulpy portions of food and the secretions, which decomposing, renderd the substitute a cesspool of impurities, alike harmful and disgusting. The use of rubber enables the operator to secure the most advantageous articulation of the teeth by an optional disposition of them in relation to the base. This is not true of the other method, since they must rest immediately in contact with the metallic plate, oftentimes necesitating a faulty inclination and consequent faulty occlusion ot the teeth, a matter of prime importance in respect to the efficiency of the substitute. In the use of rubber, great latitude is afforded in the restoration of the accustomed fullness and contour of the lips and cheeks. Not so of the other. Substitutes of rubber are more readily repaired and with less risk of injury to the teeth and impairment of adaptation. They may also be reproduced or duplicated, a thing impracticable by the other method. And so on.

What is here said in behalf of the rubber base, applies with equal propriety to celluloid. Now these are all points of superiority of these plastic, vegetable substances over gold as a base. In what respects are they inferior or less desirable? Being but indifferent conductors, they produce a certain morbid condition of the parts on which the substitutes rest, probably by retention of animal heat, but which is of so harmless a nature that the patient's attention is rarely directed to it. Local and constitutional injury is ascribed to the poisonous action of the red oxide of mercury entering into the composition of the rubber, but this, which is greatly a matter of speculation, is now being obviated by the exclusion, wholly or in large part, of this objectionable coloring ingredient. Celluloid is wholly exempt from this objectionable feature and recent improvements in its manufacture and modes of manipulation give assurance that at no distant day, its general introduction into practice will probably in a great measure supersede the use of the former.

Now when it is considered that, in connection with the use of these plastic materials, the same requirements obtain in reference to the appropriate selection of teeth for individual cases, and the same exercise of judgment and artistic taste in their arrangement, as in the older methods, we ask, in all seriousness, if these later methods of replacement do not, upon the whole, indicate improvement, rather than retrogression. If by achieving the same or better results by such processes as simplify and expedite the work of the laboratory is evidence of degeneracy in the mechanical department, then, by parity of reasoning, the use of all the modern appliances to accomplish like results at the chair, as the rubber dam, burring engine, etc., are just as conclusive evidences of degeneracy in the operative branch. As pertinent to the matter in hand, suppose some one should put himself in the way of becoming a millionaire within the next twelve months by devising a plastic material for stopping teeth which should combine perfectly all the essential and required properties of a filling, would the Dr. regard the introduction of such a substance as an evidence of retrogression in the operative department, and would he refuse, on the same grounds, to abandon the present use of gold and the more tedious, laborious and complex processes of working it. Will our distinguished friend rise and answer categorically.

The author says that within the last fifteen years there has of been an increasing demand for cheap sets of teeth, the result which is "that the better class of men entering the profession are devoting their time, almost exclusively, to operative dentistry." If this be true, commend to us cheap sets of teeth. If their cheapness leads to such a desirable result, we know of no better purpose they could serve, unless it be the satisfaction of the claim which the poorer classes, and they constitute the mass of mankind, have upon us as practitioners of a beneficent and humane calling which contemplates something more than a large margin of profits.

The truth is that practitioners are giving their attention to operative practice now more than formerly because less of their time is required in personal supervision of the tedious and complex processes of twenty years ago, and which have since been so simplified in connection with other methods, that they may now be almost exclusively entrusted to assistants. If there is an inferior class of men availing themselves of the simplified methods of this department for unworthy purposes our case is by no means phenomenal, as this evil finds its counterpart in the other professions. Medicine has its quacks, law its shysters, and divinity its imposters, but the abuse of opportunities in any of these does not by any means derogate from their character, usefulness or importance.

The author arbitrarily divides the profession in the United States into two classes, one of which embraces 2,000 competent practitioners of scientific dentistry, and the other 10,000 incompetents. The practice of the first class, he says, is established upon a medical basis, while the latter relates only to a mechanical art, and of these he remarks that "the tastes, habits and acquirements of the two classes are as divergent, as are the characters of true science and art." Now, this statement is disingenious, as there are no two classes in the sense of one being devoted exclusively to mechanical and the other to the remaining branches, but on the contrary only a single class whose practice embraces all the departments alike, and it might with equal propriety be said that the tastes, habits and acquirements of the dental practitioner who performs a purely surgical operation for the removal of a tumor, and another who performs the purely mechanical operation of filling a tooth, are as divergent as are the characters of true science and mechanism. We believe with Dr. Allport that, for obvious reasons, "the practice of both by the same individual prevents the highest development of either" but this does not by any means imply that there is any inconsistency in a dental practitioner doing so if inclination prompts him or his interests demand it.

If Dr. Allport's mode of reasoning in this connection is admitted, we shall soon have a demand for elimination in the practice of operative dentistry, and certainly it might be pressed for the same reasons. Allaying sensibility of dentine and the treatment of alveolar abscess, and of the pulp preparatory to the stopping of teeth, etc., being established upon a medical basis, and the mere mechanical operation of filling, which relates only to a mechanical art, implies the same incompatibility of taste, habits and acquirements in respect to the nature of the services rendered as is claimed to exist between the departments under consideration. We fear the measure of our author's ambition will scarcely be satisfied short of that ultimate degree of medical sublimation, so to speak, foreshadowing the professional millenium when we may not only lay unquestioned claim to the title of dontologists, but may even aspire to the still prouder distinction of being dubbed odontologists, as suggested by our gushing friend Dr. Mills, of Brooklyn, in a recent number of the St. Louis Journal.

"To drop the teaching of mechanical dentistry in private offices and in our colleges, would," we are told "in a few years, permanently divide the practice, etc."

Nothing could be more eminently Quixotic than the faith that inspired this prediction. Suppose the teaching of this department were dropped by the schools, would even any considerable number of the graduates accept it as anything binding or obligatory in practice, conflicting, as the practical separation of the departments would, with their interests. Certainly the 10,000 members of the profession in this country whom the Dr. says are not graduates, would spit upon and repudiate, as an obligation, a scheme the practical effect of which would be to jeopardize their means of support, and menace them with poverty and want, for it must be remembered that the practice of all the departments is essential to the comfortable support of a large majority of dentists and their families. The interests, as well as the inclinations of this large body of men, would combine to perpetuate the "copartnership between medical science and a mechanic art, originally entered into and conducted under the firm name of 'dentistry,' and the youngest of those now practicing it will probably never survive to see the day when the association of operative and mechanical dentistry will be 'disconnected in the minds of the public.'

"And very soon," continues the author, "each town of any considerable size, would have one or more of these (mechanical) practitioners who, by relying upon success in this calling for support, etc., would seek to redeem and elevate this particular art to the highest degree attainable." This is the most unfortunate of all the author's many false assumptions. If mechanical dentistry has retrograded, as charged, while practiced under the saving and regenerating influences of an intimate association with a learned professional body, and exposed continually to the salutary and restraining checks of its ethical regulations, to what lower depths would it sink, cut loose from such associations and professionally ostracized. Being stigmatized as a trade, it would at once sink to the level of such a pursuit, and its interests as such promoted by all the unscrupulous and nefarious tricks and devices which enter so largely into the conduct of modern competition. There would not be a square foot of blank wall, a panel of road fence, or a cross-roads finger-post that would not be plastered all over with flaring dental placards extolling the superiority of Dr. so and so's artificial teeth over those of all other manufacturers. Actuated alone by considerations of thrift in trade, there would be the strongest motives to discredit the value of all operations upon the natural teeth, and the untold and irreparable injury which would thus be inflicted upon conservative dentistry, and through it, upon the community, would be simply incalculable, and such as no humane or conscientious man could for a moment contemplate with satisfaction.

We have extended this review beyond its intended limits. In it we have endeavored to treat the arguments employed by Dr. Allport in support of his project of dismemberment with entire fairness, as well as in the spirit of the utmost friendliness and regard for the author personally. We conclude by remarking that, recognizing as we do that the whole duty of a dentist to his patients embraces everything having relation to his welfare in matters appertaining to the oral cavity, whenever we shall discover that our professional aspirations are overleaping this central idea of duty, and we are in danger of out-growing our professional clothes and are unable longer to adapt ourself to the "regulation suit," we shall proceed, as gracefully as possible, to "step down and out" and seek some more congenial pursuit.

